# Amantadine for Dyskinesias in Parkinson's Disease: A Randomized Controlled Trial

**DOI:** 10.1371/journal.pone.0015298

**Published:** 2010-12-31

**Authors:** Hideyuki Sawada, Tomoko Oeda, Sadako Kuno, Masahiro Nomoto, Kenji Yamamoto, Mitsutoshi Yamamoto, Kinya Hisanaga, Takashi Kawamura, for the Amantadine Study Group

**Affiliations:** 1 Clinical Research Center, Utano National Hospital, Kyoto City, Japan; 2 National Center for Neurological and Psychiatric Disorders, Kodaira, Japan; 3 Department of Therapeutic Medicine, School of Medicine, Ehime University, Toon, Japan; 4 Department of Neurology, Kagawa Prefectural Central Hospital, Takamatsu, Japan; 5 Department of Neurology, Miyagi National Hospital, Watari-gun, Japan; 6 Student Health Center, Kyoto University, Kyoto City, Japan; Boston University School of Medicine, United States of America

## Abstract

**Background:**

Dyskinesias are some of the major motor complications that impair quality of life for patients with Parkinson's disease. The purpose of the present study was to investigate the efficacy of amantadine in Parkinson's disease patients suffering from dyskinesias.

**Methods:**

In this multi-center, double-blind, randomized, placebo-controlled, cross-over trial, 36 patients with Parkinson's disease and dyskinesias were randomized, and 62 interventions, which included amantadine (300 mg /day) or placebo treatment for 27 days, were analyzed. At 15 days after washout, the treatments were crossed over. The primary outcome measure was the changes in the Rush Dyskinesia Rating Scale (RDRS) during each treatment period. The secondary outcome measures were changes in the Unified Parkinson's Disease Rating Scale part IVa (UPDRS-IVa, dyskinesias), part IVb (motor fluctuations), and part III (motor function).

**Results:**

RDRS improved in 64% and 16% of patients treated with amantadine or placebo, respectively, with significant differences between treatments. The adjusted odds-ratio for improvement by amantadine was 6.7 (95% confidence interval, 1.4 to 31.5). UPDRS-IVa was improved to a significantly greater degree in amantadine-treated patients [mean (SD) of 1.83 (1.56)] compared with placebo-treated patients [0.03 (1.51)]. However, there were no significant effects on UPDRS-IVb or III scores.

**Conclusions:**

Results from the present study demonstrated that amantadine exhibited efficacious effects against dyskinesias in 60–70% of patients.

**Trial Registration:**

UMIN Clinical Trial Registry UMIN000000780

## Introduction

Parkinson's disease is one of the most prevalent neurodegenerative disorders, with an increasing prevalence in the elderly [1]. Motor disturbances due to Parkinson's disease can be relieved by medications containing levodopa or dopaminergic agonists, and the majority of patients are treated with these drugs over a long period of time. Motor complications, such as dyskinesias and motor fluctuations, are often observed in long-term treated patients. Deep-brain stimulation of the subthalamus is an efficacious treatment for dyskinesias and motor fluctuations; however, this surgical procedure is invasive and indications are limited [2]. Motor complications such as dyskinesias impair quality of life and are difficult to control [3]; de-escalation of levodopa reduces dyskinesias, but is often associated with worsened motor symptoms.

Studies have suggested that dyskinesias are due to over-release of dopamine [4], hypersensitivity of striatal dopamine receptors [5], or both. Animal dyskinesia experimental models have revealed that the NR2B subunit of the *N*-methyl-d-aspartate (NMDA)-type glutamate receptor is redistributed from synaptic sites to extra-synaptic sites in the striatum [6]. The altered discharge pattern of striatal medium spiny neurons plays an important role in dyskinesias [7], and depolarization of these neurons requires glutamatergic inputs [8], [9]. Although glutamatergic inputs *via* AMPA/kainate receptors might be involved [9], synergic synaptic transmission *via* dopamine D1 receptors and NMDA receptors underlies the occurrence of dyskinesias [10].

Amantadine is a low-affinity, non-competitive antagonist of NMDA receptors [11] and is expected to ameliorate dyskinesias. Although previous studies have demonstrated that amantadine exhibits anti-dyskinetic effects [12], [13], [14], [15], [16], [17], and the duration of anti-dyskinetic effects is attenuated to within 8 months [15], the withdrawal of amantadine worsens dyskinesias, even after amantadine treatment for 1 year or longer [17]. However, the evidence for anti-dyskinetic effects of amantadine is insufficient [18], [19]. Therefore, the purpose of this study was to clarify the efficacy of amantadine in patients with dyskinesias. In addition, clinical features associated with the anti-dyskinetic effects were investigated.

## Methods

The protocol for this trial and supporting CONSORT checklist are available as supporting information; see Checklist S1 and Protocol S1. This clinical trial was designed and reported according to recommendations of the Consolidated Standard of Reporting Trials (CONSORT) statement [20].

### Study design and organization

This trial was registered in the UMIN Clinical Trial Registry (UMIN 000000780) on July 30, 2007 (https://center.umin.ac.jp/cgi-open-bin/ctr/ctr.cgi).

This multi-center, placebo-controlled, double-blinded, randomized, cross-over trial was organized by a study group comprising 13 sites in 11 prefectures in Japan. Amantadine hydrochloride was donated by Novartis Pharma Corporation, Tokyo, Japan. The study was conducted by a coordination center at Utano National Hospital and was approved by the Bioethics Committee of Utano National Hospital, the Ethica of National Center for Neurological and Psychiatric Disorders, Ehime University Hospital IRB, the Ethics Committee of Miyagi National Hospital, the Ethics Committee of Mie University Hospital, the Ethics Committee at Sagamihara National Hospital, the Ethical Committee of Research Institute for Brain and Blood Vessels Akita, the Ethical Review Committee of National Defense Medical College, the Ethics Committee of Nishitaga National Hospital, Bioethics Committee of Jichi Medical University, the Ethics Committee of Saigata National Hospital, and the Institutional Review Board of Kagawa Prefectural Central Hospital. All subjects were informed of study protocols and study relevance, and the subjects provided written consent. Safety was monitored with attention paid to adverse effects.

### Patients and eligibility

Eligible subjects were 20–75 years old and were diagnosed with Parkinson's disease (according to steps 1 and 2 of the United Kingdom Parkinson's Disease Society Brain Bank Diagnostic Criteria [21]), as well as dyskinesias of the limbs or trunk. Severity of dyskinesias was not considered as an eligibility criterion. Subjects were excluded due to the following: (1) treatment with amantadine hydrochloride in the previous two weeks; (2) psychiatric symptoms, such as auditory hallucinations or delusions (patients with a past history of visual hallucination were included); (3) estimated creatinine clearance <75 ml/min/1.73 m^2^, according to the Cockcroft-Gault formulation; (4) significant liver damage; (5) pregnancy or possible pregnancy; or (6) history of epilepsy. Patients, who met the criteria and were examined between July 2007 and August 2008, were considered for the study. The drug doses for Parkinson's disease were fixed throughout the study period.

### Randomization and treatment interventions

Patients were judged eligible by neurologists at the participating hospitals and were consented for enrollment; peripheral blood was sampled at each hospital, and creatinine clearance was calculated at the coordination center. Eligible participants were provided unique subject identification numbers according to study criteria and were assigned to Arm 1 or Arm 2 by a research technician (K.H.), according to a computer-generated, randomization plan, which included stratification by severity of dyskinesia (ADL-interfering or not-interfering). Study medications were sent to each hospital from the coordinating center, according to the schedule. A list of subject identification numbers and corresponding treatment assignments was restricted to K.H. and were concealed from other study personnel.

**Figure 1:**
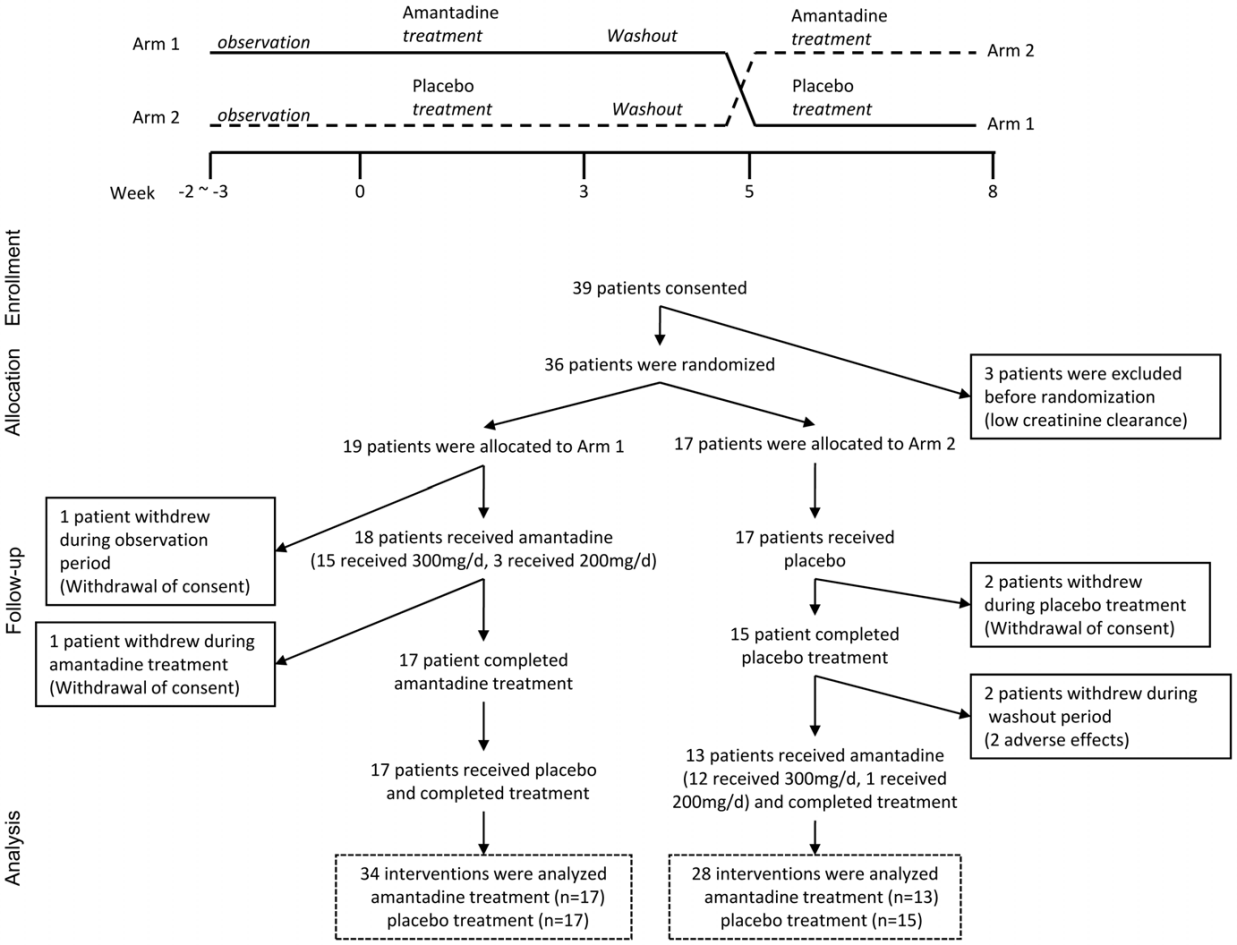
Study design and flow diagram. Top, cross-over scheme of patients randomly allocated to Arms 1 and 2. In Arm 1, amantadine was increased from 100 mg to 300 mg every 7 days, and decreased every 3 days. At 15 days after washout, placebo was administered in a similar manner. In Arm 2, placebo was increased every 7 days and decreased every 3 days, which was followed by a similar washout period and amantadine was then administered in the same fashion. Bottom, flow diagram of patients in the study.

Arm 1 intervention consisted of an observation period (2–3 weeks), amantadine hydrochloride treatment period (27 days), washout period (15 days), and placebo treatment period (27 days). Arm 2 intervention consisted of an observation period, placebo period, a washout period, and an amantadine treatment period (**Figure 1**, top). Amantadine was increased in a step-by-step manner (100 mg for 7 days, 200 mg for 7 days, and 300 mg for 7 days), followed by a decreased treatment regimen (200 mg for 3 days and 100 mg for 3 days). Placebo was also administered in a similar manner. The subjects were interviewed every 7th day, and adverse effects were monitored. Trial drugs were not increased if the patients did not desire the increase or if adverse effects were detected.

### Patient evaluations

The primary outcome measure was changes in the Rush Dyskinesia Rating Scale (RDRS) from pre-intervention time points. RDRS (from 0 absent to 4 violent dyskinesia), the inter-rater, and intra-rater reliability, which were robust [22], was used for objective evaluation of dyskinesias at the beginning and end of each intervention. Patients and the family members were instructed to video record typical dyskinesias while walking, drinking from a cup, putting on a coat, and buttoning clothing during the 3 days prior to the study visits, and RDRS scores were recorded according to the videotapes. Patients were defined as “responders” when the RDRS reduction by amantadine treatment was greater than with placebo treatment. “Non-responders” were defined when RDRS reduction by amantadine was the same or less than with placebo, and the prevalence of improvement in RDRS was compared between amantadine and placebo interventions.

The secondary outcome measures were changes in the Unified Parkinson's Disease Rating Scale-III (UPDRS-III) for motor functions (from 0 best to 116 worst), UPDRS-IVa for dyskinesia (from 0 absent to 13 worst) and UPDRS-IVb for motor fluctuations (from 0 best to 7 worst). UPDRS-III was administered in the ON period in patients with motor fluctuations. UPDRS-I, II, and IV were assessed based on interviews with patients, and UPDRS-III was evaluated according to findings of study examinations.

The relationships between response to amantadine and clinical features, including subject characteristics (age, sex, duration of disease, and age of onset), amantadine dose, amantadine plasma concentrations, and doses of anti-Parkinson's drugs (levodopa, dopamine agonists, and entacapone) were compared. Dopamine agonist doses were calculated as the levodopa equivalent dose (LDED) [23]. Plasma concentrations of amantadine were determined as previously reported [24] and were measured at 3–6 h after administration of the morning amantadine dose.

### Sample size

Based on previous reports, dyskinesia scores were estimated to improve by −1.1 [(1.6) mean (SD)] points on the UPDRS-IVa following amantadine hydrochloride treatment [14]. Sample sizes were determined by power analysis comparing the two means and were calculated as 30 (60 interventions) to achieve a power>80% and an error of 0.05 in the cross-over test.

### Statistics

Changes in RDRS scores were regarded as ordinal variables and were categorized into two levels [improved (changes in RDRS<0), and not-improved (changes in RDRS≥0)]. Binominal generalized estimating equations with unstructured correlation matrix were adapted to fit a repeated measure logistic regression, incorporating treatment effects (amantadine or placebo), period effects (interaction of order effect and carry-over effect), and sex as main effect factors, as well as pre-treatment UPDRS-IVa scores as a covariate. The prevalence of improved RDRS was compared between amantadine and placebo treatments. The odds-ratio for improved RDRS following treatments was calculated according to the generalized estimating equation. RDRS score changes from baseline were regarded as ordinal variables, and generalized estimating equations with unstructured correlation matrix were adopted to fit repeated measure ordinal logistic regression incorporating treatment effects (amantadine or placebo) and period effects (interaction of order effect and carry-over effect), with sex as the main effect factor and pre-treatment RDRS scores as a covariate.

UPDRS score changes were regarded as scale variables. Data were analyzed using a mixed linear model, with correlated residuals assuming treatment effects, period effects, and sex as fixed-effects factors, pre-treatment scores as covariance, and interventions (first or second) as repeated-effects factors. The adjusted mean difference in scores was compared between amantadine and placebo treatments, and the direct treatment and period effects were statistically analyzed.

Clinical factors associated with a response to amantadine were analyzed using multivariate logistic regression models (backward step-wise model with a likelihood ratio test). All statistical analyses were performed using SPSS Statistics 17.0. A *P*-value<0.05 was considered statistically significant.

## Results

### Patient enrollment

Of the 39 patients identified as potential participants, three were excluded due to low creatinine clearance. The remaining 36 patients were randomized, with 19 and 17 patients allocated to Arms 1 and 2, respectively. In Arm 1, one patient withdrew consent prior to intervention, and 18 received amantadine. During amantadine treatment, one patient withdrew consent, and the remaining 17 patients received placebo and completed the study. In Arm 2, all participants received placebo, but two patients withdrew consent during placebo treatment, and two patients discontinued the study due to adverse events (one exhibited worsening dyskinesia, and one fell and experienced a fracture) during the washout period. The remaining 13 participants received amantadine and completed the study. Data from 30 amantadine interventions (17 in Arm 1, and 13 in Arm 2) and 32 placebo interventions (17 in Arm 1, and 15 in Arm 2), as well as 32 participants, were analyzed in a full analysis set (**Figure 1**). The two treatment groups were similar at baseline with respect to demographic and clinical variables (**Table 1**).

**Table 1:** Characterization of study participants.

	Arm 1	Arm 2
	amantadine to placebo	placebo to amantadine
Characteristics	(n = 18)	(n = 17)
Age, mean (SD), y	63.9 (7.6)	62.0 (7.0)
Male, No. (%)	6 (33.3)	4 (23.5)
Duration of PD, mean (SD), y	13.5 (4.5)	13.3 (9.1)
L-Dopa, mean (SD), mg/day	447 (139)	435 (171)
LDED of dopamine agonists, mean (SD), mg/day	176 (108)	151 (129)
UPDRS-III, mean (SD), points	16.7 (14.0)	22.4 (8.6)
UPDRS-IV, mean (SD), points	8.0 (3.6)	7.4 (3.1)
RDRS, median (interquartile range), points	2.0 (1.25)	2.0 (0.0)

### Efficacy measurements

**Figure 2:**
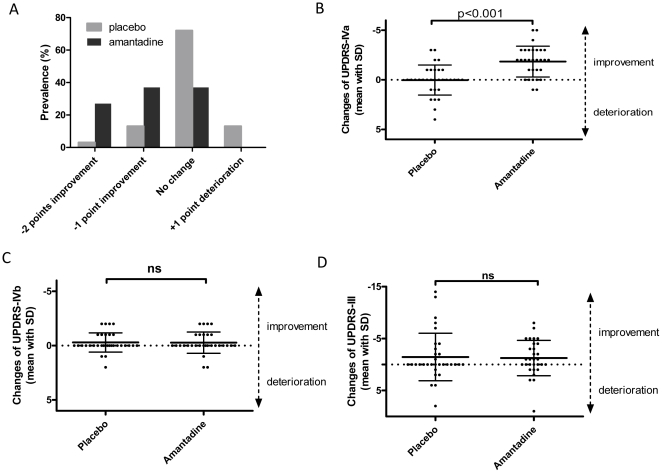
Score changes in RDRS, UPDRS-IVa (dyskinesias), IVb (motor fluctuation), and III (motor disturbance) following amantadine and placebo treatment. Following amantadine treatment, RDRS scores improved in 64% of participants (−2 points in 27%, and −1 point in 37%), but remained unchanged in 37% of participants. RDRS scores improved in 16% of participants, but did not improve in 84%, following placebo treatment (A). UPDRS-IVa scores significantly improved following amantadine treatment (B). In contrast, UPDRS-IVb and III scores did not improve following treatment with amantadine or placebo (C, D). Data are plotted as scattered diagrams and bars represent means with standard deviations of raw data.

Following amantadine treatment, RDRS scores improved in 64% of participants, and placebo treatment resulted in improvement in 16% of participants (**Figure 2A**). Statistical analysis utilizing generalized estimating equations revealed a statistically significant difference in prevalence of improvement in RDRS between amantadine and placebo treatments (*P* = 0.016), although the period effect was not statistically significant (*P* = 0.31). The odds-ratio of improvement by treatment was 6.7 [95% confidence interval (CI), 1.4 to 31.5] following adjustment for period effects. RDRS changes from baseline are shown in **Table 2**, with significant differences between treatment (*P* = 0.002, repeated measure ordinal logistic regression model using generalized estimating equations). (See Videos S1 and S2; typical patient presenting dyskinesia.)

**Table 2:** RDRS score changes in amantadine and placebo treatment.

Treatment	Change of RDRS				Treatment effect	
	−2 pt	−1 pt	0 pt	+1 pt	Adjusted OR (95% CI)*	*p*
amantadine, n (%)	8 (26.7)	11 (36.7)	11 (36.7)	0 (0.0)	10.4 (2.0 to 47)	0.002
placebo, n (%)	1 (3.1)	4 (12.5)	23 (71.9)	4 (12.5)		

*Odds ratio was adjusted for sex, period effect, pretreatment RDRS score, in an ordinary logistic regression model using general estimating equations.

n: number of interventions.

The unadjusted changes of UPDRS-IVa, IVb, and III from baseline, as well as the adjusted differences between amantadine and placebo interventions, are shown in **Table 3**. There was no period effect in score changes, and UPDRS-IVa scores improved by a mean (SD) of 1.83 (1.56) following amantadine treatment and 0.03 (1.51) following placebo treatment (**Figure 2B**). There was a statistically significant treatment effect (*P*<0.001), and the adjusted difference was a mean (95% confidence intervals) of 2.02 (1.22–2.83). UPDRS-IVb and III scores remained unchanged following amantadine or placebo treatment (**Figure 2C, D**) with no significant treatment effect on changes (UPDRS-IVb: *P* = 0.87, and UPDRS-III: *P* = 0.26). These results were identical when results from the first intervention only were analyzed to avoid carry-over effects (**Table S1**).

**Table 3:** Score changes in amantadine and placebo interventions.

	Unadjusted		Adjusted difference*		
	Amantadine	Placebo		*P* Value	
	(n = 30)	(n = 32)		treatment effect	period effect
change of UPDRS-IVa, mean (SEM)	−1.83 (0.28)	0.03 (0.27)	−2.02 (0.39)	<0.001	0.48
change of UPDRS-IVb, mean (SEM)	−0.27 (0.18)	−0.28 (0.16)	0.05 (0.28)	0.87	0.77
change of URDRS-III, mean (SEM)	−1.23 (0.62)	−1.43 (0.81)	1.85 (1.60)	0.26	0.23

*Difference of score changes (negative values indicate improvement) was adjusted for sex, period effect, pretreatment scores using a mixed linear model.

n: number of interventions.

### Safety analysis

Adverse events were observed in nine patients (six receiving amantadine, one receiving placebo, and two during washout). The most common adverse effect was visual hallucinations, which was observed in three patients during the amantadine treatment period. The prevalence of adverse effects was significantly greater in patients receiving amantadine treatment compared with placebo treatment (*P* = 0.048) (**Table 4**).

**Table 4:** Adverse effects in the study.

	placebo	amantadine	
	(n = 34)	(n = 31)	*P* Value
visual hallucination, No. (%)	0 (0.0)	3 (9.7)	
blurred vision, No. (%)	0 (0.0)	1 (3.2)	
constipation, No. (%)	0 (0.0)	1 (3.2)	
fall, No. (%)	0 (0.0)	1 (3.2)	
worsening of AIM, No. (%)	1 (2.9)	0 (0.0)	
worsening of off-phenomenon, No. (%)	1 (2.9)	0 (0.0)	
total, No. (%)	1 (2.9)	6 (19.4)	0.048

Fisher's exact test.

### Clinical features associated with anti-dyskinetic effects

Of the 30 participants who completed the study, 20 patients responded to amantadine. The demographical (age and sex) and clinical features [onset age of Parkinson's disease, dose of L-Dopa, entacapone, and dopamine agonist (LEDD), dyskinesia severity (pretreatment UPDRS-IVa) and plasma concentration of amantadine] were included for analysis using multivariate logistic regression models. Results showed that patients with a higher age of Parkinson's disease onset (odds-ratio = 5.9 (95% confidence interval, 1.1–32.6, *P* = 0.04)/10 years) and higher doses of dopamine agonists (odds-ratio = 10.0 (1.2–81.3)/100mg LDED) were more likely to respond to amantadine.

## Discussion

The anti-dyskinetic effects of amantadine have been previously evaluated in six studies (three parallel [12], [15], [17] and three cross-over [13], [14], [16] studies). Although a cross-over design study has the advantage that the sample size could be reduced, period effects, including carry-over effect, cannot be neglected. In the present study, the treatment effect was evaluated following statistical adjustment for period effects and amantadine-improved dyskinesias. Anti-dyskinetic effects were confirmed in both RDRS and UPDRS-IVa. As shown in **Table 2**, RDRS decreased by 1 or 2 points in 63.4% of patients following amantadine treatment, and the changes reached a clinically meaningful level, because RDRS is scored according to interference with function of voluntary movement or daily activities. The adjusted treatment effect in UPDRS-IVa was estimated at 2.02, which was consistent with previous results [12], [13], [14], [15], [16]. Though it is important to evaluate the quality-of-life or cost-benefit ratios with amantadine therapy, these data were not obtained because the original aim of this study was to investigate the efficacy of anti-dyskinetic effects.

UPDRS-III (motor disturbance) was not altered by amantadine treatment, which was consistent with previous studies [13], [14], [16], [17]. Motor disturbance effects have been shown to be masked by a sufficient dose of L-dopa and dopamine agonists during advanced stages of disease [13].

Results demonstrated that amantadine ameliorated dyskinesias in 20 of 30 patients, but was not efficacious in the remaining 10 patients. Multivariate logistic analysis revealed that higher age-of-onset and use of dopamine agonists positively associated with the response to amantadine. Because dyskinesias are more often observed and are more severe in young-onset PD patients compared with elderly-onset patients [25], amantadine might not suppress severe dyskinesias in younger patients. However, the severity of dyskinesia was not identified as an associated factor in the present study. Previous results have shown that transient dyskinesia observed immediately following subthalamotomy is not ameliorated by amantadine [26]. Therefore, activity in the subthalamic nuclei could change with age and use of dopamine agonists. However, further studies are needed to determine the precise mechanisms underlying dyskinesias in non-responders.

### Conclusions

Results from the present study demonstrated that amantadine was efficacious for dyskinesias in 60–70% of patients in advanced stages of Parkinson's disease.

## Supporting Information

Checklist S1 **CONSORT Checklist.**
(DOC)

Protocol S1 **Trial Protocol.**
(PDF)

Video S1 **Typical dyskinesias recorded from a patient at Visit 2 (before the treatment).** Dyskinesias in limbs and trunk impaired activity of daily living are shown.
(MOV)

Video S2 **Typical dyskinesias recorded from the same patient at Visit 5 (amantadine, 300mg/d).** Dyskinesias were markedly improved. Videos S1 and S2 were recorded at exactly the same time of day.
(MOV)

Table S1 **Changes of UPDRS scores in 1st intervention are shown in the supplemental table.**
(PDF)
