# Supplementary material for: Amantadine for Dyskinesias in Parkinson's Disease: A Randomized Controlled Trial
Source: PLoS One. 2010 Dec 31;5(12):e15298. doi: 10.1371/journal.pone.0015298 (PMC3013111; doi:10.1371/journal.pone.0015298)
Supplement: Protocol S1 — Trial Protocol. (PDF) [file pone.0015298.s002.pdf]

A Randomized, Double-blind, Placebo-controlled, Cross-over, Multi-center Study Concerning the Efficacy and Safety of Amantadine Hydrochloride in Treatment of Dyskinesias in Parkinson's Disease

STUDY PROTOCOL

[Purpose of the study]

To make clear whether amantadine hydrochloride improves dyskinesias without worsening motor function by a multi-center, randomized, double-blind trial.

[Background of the study]

The prevalence of Parkinson's disease (PD) increases in accordance with aging, and PD is one of the most important brain diseases in Japan. Motor disturbance of PD is caused by mainly degeneration of dopaminergic neurons in the central nervous system, and dopaminergic replacement therapy using L-Dopa or dopamine receptor agonists improves motor symptoms. As a result, an increasing number of patients are treated with dopaminergic replacement therapy for a long time. In a long time therapy there are several issues that include dyskinesias. Dyskinesias are thought to be related to hypersensitivity of the dopaminergic receptors in the striatum and the excessive release of dopamine from the dopaminergic neurons, and in addition, it could be related to stimulation to the N-methyl-D-aspartate (NMDA) type glutamate receptors. Amantadine hydrochloride is approved for treatment against PD. Pharmacological actions of amantadine are dopamine release enhancement, dopamine reuptake inhibition, and in addition, amantadine blocks the NMDA type glutamate receptor. Dopamine release from the striatal dopaminergic neurons is regulated via the NMDA-glutamate receptor, and therefore, amantadine may block dyskinesias. Because the concentrations of dopamine and its metabolites in the brain are not influenced by amantadine in the doses of clinical usage its anti-Parkinsonian effects are thought to be related to its anti-cholinergic action or to blockade of the NMDA-glutamate receptor.
In the Treatment Guideline 2002 for Parkinson's disease released from the Japan Neurological Society, the cease of MAO-B inhibitors or de-escalation of L-Dopa are recommended. However the cease of MAO-B inhibitors and L-Dopa de-escalation cause worsening of motor symptoms of PD, and the treatment against dyskinesias without worsening of motor symptoms is preferred. The purpose of the study is to clarify whether or not amantadine improves dyskinesias without worsening of motor function by a multi-center, randomized, double-blind trial.

[Methods]

1. The study design

This is a multi-center, randomized, placebo-controlled, double-blind, cross-over study. A cross-over design is adopted to avoid disadvantage in subjects who are allocated in the placebo treatment because amantadine has beneficial effects against PD.

2. Eligibility

Eligible patients are 20 to 75 years-old, diagnosed as PD according to steps 1 and 2 of U.K. Parkinson's disease Society Brain Bank Diagnostic Criteria and presenting dyskinesias of the limbs or the trunk.

3. Exclusion criteria

1) Patients prescribed with amantadine hydrochloride during the previous 2 weeks
2) Patients with psychiatric symptoms such as auditory hallucination or delusions
3) According to the following formula, creatinine clearance is less than 75mL/min/1.73m2
Male : (140-age) x weight(kg)/(72 x serum creatinine)(mg/dL)
Female : (140-age) x weight(kg) x 0.85/(72 x serum creatinine)(mg/dL)
4) Remarkable liver damage
5) Pregnant or possibly pregnant
6) History of epilepsy
7) Patients who are judged as inappropriate participants in the trial

4. Informed consent

Patients who are eligible and not excluded according to the exclusion criteria described above will be informed of the background, purpose, method, spontaneous participation, withdraw rights of the study, and gave written consent. Study subjects can read this protocol if necessary.

5. Privacy of study subjects

Data obtained in the study will be handled anonymously.

6. Interventions

Patients who meet the criteria and seen between March 2007 and February 2008[*1] were considered for the study.

The Arm 1 intervention consisted of an observation period (2-3 weeks), an administration of amantadine hydrochloride period (27 days), a washout period (15 days), and an administration of placebo period (27 days). The Arm 2 intervention consisted of an observation period, placebo period, a washout period, and an administration of amantadine period. Amantadine was escalated step-by-step (100 mg for 7 days, 200 mg for 7 days and 300 mg for 7 days). Placebo is also administered in a

similar manner. The detailed schedule is shown in the Table 1.

Table 1. Schedule of the study

Schedule of the study

			Visit	UPDRS /Rush dyskinesia scale	blood sampling	video recording	Arm 1	Arm 2
Visit 1	Day -13		○		○			
	Day -12							
	Day -11							
	Day -10							
	Day -9							
	Day -8							
	Day -7						Observation period (2-3 W)	Observation period (2-3 W)
	Day -6							
	Day -5							
	Day -4							
	Day -3							
	Day -2					○		
	Day -1							
Visit 2	Day 0	±3D	○	○				
	Day 1							
	Day 2							
	Day 3						Active 1.0g /1x1	Placebo 1.0g /1x1
	Day 4							
	Day 5							
	Day 6							
visit 3	Day 7	±3日	(○)					
	Day 8							
	Day 9							
	Day 10							
	Day 11						Active 2.0g /2xN	Placebo 2.0g /2xN
	Day 12							
	Day 13							
Visit 4	Day 14	±3日	(○)					
	Day 15							
	Day 16							
	Day 17							
	Day 18						Active 3.0g /3xN	Placebo 3.0g /3xN
	Day 19					○		
	Day 20							
Visit 5	Day 21	±3日	○	○	◎			
	Day 22							
	Day 23						Active 2.0g /2xN	Placebo 2.0g /2xN
	Day 24							
	Day 25						Active 1.0g /1x1	Placebo 1.0g /1x1
	Day 26							
	Day 27							
	Day 28							
	Day 29							
	Day 30							
	Day 31							
	Day 32							
	Day 33							
	Day 34							
	Day 35						Washout period (2W)	Washout period (2W)
	Day 36							
	Day 37							
	Day 38							
	Day 39							
	Day 40					○		
	Day 41							
Visit 6	Day 42	±3日	○	○	◎			
	Day 43							
	Day 44							
	Day 45						Placebo 1.0g /1x1	Active 1.0g /1x1
	Day 46							
	Day 47							
	Day 48							
Visit 7	Day 49	±3日	(○)					
	Day 50							
	Day 51							
	Day 52						Placebo 2.0g /2xN	Active 2.0g /2xN
	Day 53							
	Day 54							
	Day 55							
Visit 8	Day 56	±3日	(○)					
	Day 57							
	Day 58							
	Day 59						Placebo 3.0g /3xN	Active 3.0g /3xN
	Day 60							
	Day 61					○		
	Day 62							
Visit 9	Day 63	±3日	○	○	◎			
	Day 64							
	Day 65						Placebo 2.0g /2xN	Active 2.0g /2xN
	Day 66							
	Day 67						Placebo 1.0g /1x1	Active 1.0g /1x1
	Day 68							
	Day 69							
Visit 10	Day 70	±3日	○					
	Day 71							
	Day 72							
	Day 73							
	Day 74							
	Day 75							

◎ plasma concentration of amantadine
○ CBC, Chemical analysis
(○) on site investigation or telephone contact

Escalation of trial drugs could be discarded if the patients desired no escalation or adverse effects were detected.

Allocation to Arm 1 or Arm 2 is determined according to a computer-generated randomization plan that includes stratification by severity of dyskinesia (ADL-interfering or not-interfering).

At the end of the observation period and at the day 21 of the amantadine or placebo treatment periods, UPDRS-3, UPDRS-4, video recording of dyskinesias and Rush Dyskinesia Rating Scale[*2] (RDRS), medication, blood sampling, and type of dyskinesias (peak dose, end-of-dose, or biphasic dyskinesia) will be checked. Plasma concentrations of amantadine hydrochloride will be determined at the day 21 of amantadine or placebo periods and at the end of washout period.

7. Target sample size

Target sample size is 60 interventions. The sample size was determined based on the previous study by Snow et al., in which dyskinesia score is expected to be improved by 1.1 (1.6) (mean (SD) points on the UPDRS-IVa by amantadine. It was estimated by power analysis comparing the two means, and calculated as 30 (60 interventions) to achieve a power greater than 80% and an error of 0.05 in the crossover test.

8. Administration of the investigatory product (IP)

Amantadine or placebo will be administered according to the schedule table shown in Table 1.

9. Investigatory product (IP)

Placebo consists of 1.0g of lactate and 0.1g of $MgCl_2$. Active consists of 1.0g of 10% amantadine hydrochloride and 0.1g of $MgCl_2$.

10. Randomization

Randomization is based on a computer-generated plan that was concealed.

11. Limitation and fixation of concomitant medications

For the purpose of the study concomitant Parkinson's disease medications shown in the List 1 will be fixed during the course of the study. Medications in the List 2 will be fixed during the course of the study. Use of amantadine is prohibited. Setting of the deep brain stimulation (DBS) should not be changed during the study period in patients with DBS surgical operation.

List 1. (Concomitant medications for PD)

Levodopa (Dopa, Dopa with carbidopa, Dopa with benzeraside)

Agonists for the dopamine receptor (bromocriptine, pergolide, cabergoline, talipexole, pramipexole, ropinirol)

selegiline

zonisamide

trihexyphenidyl

List 2 (Anti-psychotic drugs with dopamine receptor inhibition)

tiapride, sulpiride, quetiapine, chlorpromazine, thioridazine, fluphenazine, fludecasin, prochlorperazine, propericiazine, levomepromazine, haloperidol

12. Analysis

Primary outcome measure:

Primary outcome measure is the difference of Rush Dyskinesia Scale from baseline after amantadine hydrochloride and after placebo administration.

The changes of scores from baseline will be compared between amantadine and placebo interventions.

Changes of RDRS will be categorized into improved and not-improved, and data will be analyzed using repeated measure logistic regression incorporating treatment effects (placebo vs active) and period effects (interaction of order effect and carry-over effect).

P values of less than 0.05 are considered statistically significant.

Rush Dyskinesia Scale is scored based on video recording of dyskinesias.

Secondary outcomes:

Secondary outcome measures include the difference of UPRDS-III, IVa, and IVb from baseline after amantadine hydrochloride and after placebo administration. The changes of score from baseline will be compared between amantadine and placebo interventions.

UPDRS-III is scored on neurological examination performed by neurologists

UPDRS-IV is scored by interview.

Changes of UPDRS-IVa will be analyzed using a mixed linear model incorporating treatment effects (placebo vs active) and period effects (interaction of order effect and carry-over effect).

In addition secondary outcome measures include the correlation between blood concentrations of amantadine hydrochloride and the UPDRS (parts III and IV) and Rush Dyskinesia Scale changes.

Safety analysis

The prevalence of adverse events will be compared between amantadine and placebo interventions.

[Response to adverse events]

In case of adverse events escalation of the IP should be discarded, the IP should be de-escalated or ceased if necessary. Adverse events should be reported to the principal investigator and the study supervisor.

[Handling of study data]

Data will be collected and fixed by the principal investigator. Data will be analyzed after key-open.

[Registration of the study]

The study will be registered in UMIN-Clinical Trials Registry (UMIN-CTR).

[Publication of the study]

The study will be published to the journal under the names of the investigators, or the name of study group if necessary.

ADDENDUM 1.

---Video recording instruction---

Please record dyskinesia (involuntary movement) as follows:

1. Record the patient walking (for 10 meters).
2. Record the patient drinking from a cup.
3. Record the patient putting on a coat and button clothing.

Please record the movement of the head, trunk and upper and lower extremities of patients.

ADDENDUM 2

This protocol has been approved by the ethical review board of National Utano Hospital, National Hospital Organization (18-15) on March 1, 2007, and then approved by the institutional ethics review board at each participating site.*

* the Bioethics Committee of Utano National Hospital, the Ethics Committee of National Center for Neurological and Psychiatric Disorders , Ehime University Hospital IRB, the Ethics Committee of Miyagi National Hospital, the Ethics Committee of Mie University Hospital, the Ethics Committee at Sagamihara National Hospital, the Ethical Committee of Research Institute for Brain and Blood Vessels Akita, the Ethical Review Committee of National Defense Medical College, the Ethics Committee of Nishitaga National Hospital, Bioethics Committee of Jichi Medical University, and the Ethics Committee of Saigata National Hospital, and the Institutional Review Board of Kagawa Prefectural Central Hospital
