# Supplementary material for: Amantadine for Dyskinesias in Parkinson's Disease: A Randomized Controlled Trial
Source: PLoS One. 2010 Dec 31;5(12):e15298. doi: 10.1371/journal.pone.0015298 (PMC3013111; doi:10.1371/journal.pone.0015298)
Supplement: Table S1 — Changes of UPDRS scores in 1st intervention are shown in the supplemental table. (PDF) [file pone.0015298.s005.pdf]

Supplementary table. Changes of scores in 1st intervention

	Placebo (n=15)	Amantadine (n=17)	*P* Value*
UPDRS-IVa, mean (SEM)	-0.33 (0.21)	-2.35 (0.32)	<0.001
UPDRS-IVb, mean (SEM)	-0.40 (0.19	-0.35 (0.19)	0.86
UPDRS-III, mean (SEM)	-2.33 (1.38)	-0.47 (0.84)	0.25

* t-test
